# Research on emotion recognition using sparse EEG channels and cross-subject modeling based on CNN-KAN-$F^{2}CA$ model

**DOI:** 10.1371/journal.pone.0322583

**Published:** 2025-05-27

**Authors:** Fan Xiong, Mengzhao Fan, Xu Yang, Chenxiao Wang, Jinli Zhou

**Affiliations:** 1 Zhongyuan University of Technology, Zhengzhou, China; 2 Shengda Economics Trade and Management College of Zhengzhou, Zhengzhou, China; Nanyang Technological University, SINGAPORE

## Abstract

Emotion recognition plays a significant role in artificial intelligence and human-computer interaction. Electroencephalography (EEG) signals, due to their ability to directly reflect brain activity, have become an essential tool in emotion recognition research. However, the low dimensionality of sparse EEG channel data presents a key challenge in extracting effective features. This paper proposes a sparse channel EEG-based emotion recognition method using the CNN-KAN-$F^{2}CA$ network to address the challenges of limited feature extraction and cross-subject variability in emotion recognition. Through a feature mapping strategy, this method maps features such as Differential Entropy (DE), Power Spectral Density (PSD), and Emotion Valence Index (EVI) - Asymmetry Index (ASI) to pseudo-RGB images, effectively integrating both frequency-domain and spatial information from sparse channels, providing multi-dimensional input for CNN feature extraction. By combining the KAN module with a fast Fourier transform-based $F^{2}CA$ attention mechanism, the model can effectively fuse frequency-domain and spatial features for accurate classification of complex emotional signals. Experimental results show that the CNN-KAN-$F^{2}CA$ model performs comparably to multi-channel models while only using four EEG channels. Through training based on short-time segments, the model effectively reduces the impact of individual differences, significantly improving generalization ability in cross-subject emotion recognition tasks. Extensive experiments on the SEED and DEAP datasets demonstrate the proposed method’s superior performance in emotion classification tasks. In the merged dataset experiments, the accuracy of the SEED three-class task reached 97.985%, while the accuracy for the DEAP four-class task was 91.718%. In the subject-dependent experiment, the average accuracy for the SEED three-class task was 97.45%, and for the DEAP four-class task, it was 89.16%.

## Introduction

Emotion recognition plays an increasingly important role in artificial intelligence and human-computer interaction. With the deepening of affective computing research, accurately identifying and interpreting human emotions has become a key issue. Electroencephalography (EEG) signals, as a bioelectric signal reflecting brain activity, have been widely applied in emotion recognition research due to their non-invasive nature and high temporal resolution. Compared to facial expressions, speech, and other physiological signals, EEG signals provide more direct neural information; however, their high dimensionality and complexity present challenges for emotion recognition [1] .

In order to effectively utilize EEG signals for emotion recognition, researchers have attempted to extract various features from the frequency, time, and time-frequency domains. However, how to reduce redundancy and extract effective emotional features from sparse EEG channels remains a challenge. Significant progress has been made in current emotion recognition research, particularly in the area of EEG-based emotion recognition. Researchers have proposed various methods to process and analyze EEG data to improve the accuracy of emotion classification.

Traditional machine learning methods, such as Support Vector Machines (SVM), K-Nearest Neighbors (KNN), and Linear Discriminant Analysis (LDA), were widely used in early EEG emotion recognition studies. These methods rely on manually extracted features, such as Power Spectral Density (PSD), Differential Entropy (DE), Event-Related Variability, etc. These features are extracted during the data preprocessing stage from EEG signals and are used as input for classifiers in emotion classification [2–5]. Prakash *et al*. systematically compared the performance of various machine learning methods, including SVM, decision trees, and XGBoost, in EEG tasks, further validating their applicability and limitations in different datasets [6]. These manually extracted features effectively leverage domain knowledge and offer advantages in terms of computational efficiency and model interpretability. However, the performance of these methods is highly dependent on the quality and quantity of the extracted features and may exhibit limitations when handling complex, high-dimensional data.

In recent years, the introduction of deep learning techniques has opened new avenues for emotion recognition, especially Convolutional Neural Networks (CNN), Long Short-Term Memory networks (LSTM), and Graph Convolutional Networks (GCN), which have shown superiority in handling EEG time-series data [7–10]. With the development of these advanced technologies, the accuracy and efficiency of emotion recognition have been significantly improved. However, most current studies rely on multi-channel EEG data, but in practical applications, considering the portability of the device and user comfort, there is a growing demand for emotion recognition using sparse channels. Therefore, how to effectively extract emotional features from a limited number of channels has become a key challenge that needs to be addressed.

In response to the challenges of sparse EEG channel emotion recognition tasks and the impact of cross-subject feature differences, this paper proposes an emotion recognition model based on the CNN-KAN-$F^{2}CA$ architecture. The main contributions of this paper are as follows:

1. Feature Mapping Strategy: By mapping features such as DE, PSD, EVI-ASI, etc., into pseudo-RGB images, the model effectively integrates both frequency-domain and spatial information from sparse channels, providing multi-dimensional input for subsequent CNN feature extraction.

2. $F^{2}CA$ Attention Mechanism Design: A frequency channel attention mechanism ($F^{2}CA$) based on Fast Fourier Transform (FFT) is introduced, which adaptively focuses on key features in each frequency band and channel, enhancing the accuracy of feature representation and improving the performance of emotion recognition in sparse channels.

3. KAN Network Replacing Fully Connected Strategy: The model innovatively uses Kolmogorov-Arnold Networks (KAN) to replace traditional fully connected layers, capturing the complex relationships between features through nonlinear mapping, thus improving the abstraction capability and classification performance of emotional features, especially in sparse data environments.

4. Cross-Subject Training Based on Discretized Segments: The model adopts a training strategy based on discretized segments, enabling it to focus on capturing short-term emotional features and avoid over-reliance on unique patterns in individual brain signals. Combined with CNN’s local feature extraction capability, the model can effectively identify common features in emotions, reducing the impact of individual differences, thereby enhancing the model’s robustness and generalization ability in cross-subject emotion recognition tasks.

The remainder of this paper is organized as follows: section “Related work” provides a brief review of previous emotion recognition methods; section “Methods” presents the proposed emotion recognition method and its key components, including the overall CNN-KAN-$F^{2}CA$ network model, feature extraction and individual difference correction methods, feature mapping strategy,$F^{2}CA$ attention mechanism, and KAN module; section “Experimental design” describes the public datasets, experimental parameter settings, and evaluation metrics used in the experiments; section “Results and analysis” provides a detailed analysis of the experimental results of CNN-KAN-$F^{2}CA$ on the DEAP and SEED datasets. Section “Discussion” discusses the limitations of the proposed method and future research directions. Finally, section “Conclusion” concludes the paper.

## Related work

Emotion recognition methods are mainly divided into two categories: one based on non-physiological signals, such as facial expressions and speech, and the other based on physiological signals, such as EEG, skin conductance signals (GSR), and other peripheral physiological signals [11]. Compared with non-physiological signals, physiological signals are not subject to subjective human control and can more accurately reflect an individual’s emotional state, thus becoming an important research direction in affective computing.

Currently, research on emotion recognition based on physiological signals mainly relies on multi-channel EEG signals from the entire brain region. For example, Yin *et al*. [12] fused Graph Convolutional Networks (GCN) with Long Short-Term Memory networks (LSTM) and used 32 channels on the DEAP dataset to achieve 90.45% accuracy for arousal classification and 90.60% for valence classification. Similarly, Liu *et al*. [13] applied the GLFNet model, using 32 EEG channels to achieve 94.91%, 94.53%, and 92.92% accuracy in arousal, valence, and arousal-valence classification, respectively. Chen *et al*. [14] extracted spatial connectivity information from EEG signals and combined domain adaptation methods, achieving 95.15% binary classification accuracy on the DEAP dataset. Han *et al*. [15] proposed a multi-scale emotion recognition method (MS-ERM), which performed spatial mapping and temporal feature extraction from EEG signals using TimesNet, achieving 91.31% accuracy for arousal and 90.45% for valence classification on the DEAP dataset.

In summary, these studies mainly rely on 32 EEG channels from the entire brain region for emotion recognition. While multi-channel EEG provides rich emotional information and achieves high classification accuracy, it also leads to information redundancy, which affects the inference speed of emotion recognition and limits its application in wearable devices.

With the continuous advancement of wearable technology, miniaturized physiological signal acquisition devices based on fewer electrodes are gradually becoming popular, making emotion recognition research based on limited physiological signal channels increasingly important [16]. However, current research on sparse EEG channels still shows insufficient classification accuracy. For example, Jie *et al*. [3] proposed an emotion recognition method based on SVM, using 5 EEG channels, achieving 79.11% accuracy in arousal classification and 64.47% in valence classification. Mohammadi *et al*. [4] applied a KNN classifier and used 10 EEG channels, achieving a maximum classification accuracy of 84.05% for arousal and 86.75% for valence. Mert *et al*. [17] applied Empirical Mode Decomposition (EMD) and Multivariate Extended (MEMD) processing to EEG signals, achieving 75% arousal classification accuracy and 72.87% valence classification accuracy using 18 channels from the DEAP dataset. Additionally, Bazgir *et al*. [18] used Discrete Wavelet Transform (DWT) to decompose EEG signals into four frequency bands and extract spectral features, achieving 91.3% arousal and 91.1% valence classification accuracy using 10 channels from the DEAP dataset. Hector *et al*. [19] constructed a feature matrix based on Power Spectral Density (PSD) features using 14 channels and proposed a CNN model called BioCNN, achieving 83.12% accuracy in arousal classification and 76.78% in valence classification.

## Methods

### CNN-KAN-$F^{2}CA$ network model

**Fig 1:**
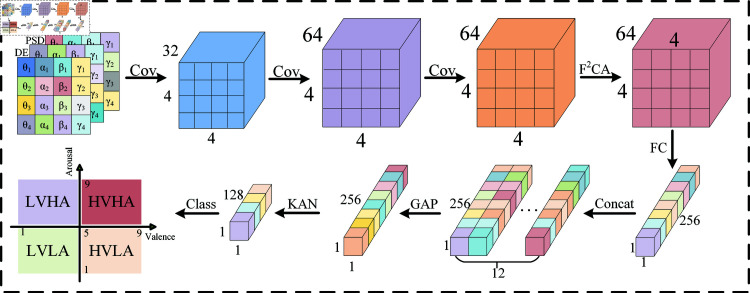
Model architecture. The samples extract deep features through multiple convolutional layers, are processed by the attention mechanism and KAN network, and are finally classified for output. Despite the progress made by these sparse-channel EEG emotion recognition methods, they generally face the issue of lower emotion recognition accuracy, significantly lower than the classification accuracy achieved by multi-channel EEG. Therefore, we attempt to develop a new emotion recognition method based on deep learning models to improve the accuracy of emotion classification.

This paper presents a sparse channel EEG emotion recognition model called CNN-KAN-$F^{2}CA$, designed to effectively extract features and perform emotion classification from limited EEG channels. The model integrates Convolutional Neural Networks (CNN), Kolmogorov-Arnold Networks (KAN), and $F^{2}CA$ to achieve efficient fusion of frequency domain information and spatial features. The overall architecture of the model is shown in Fig 1.

First, the raw signals from the sparse EEG channels undergo preprocessing to generate a pseudo-RGB image with a size of 4×4×3. This image maps DE, PSD, and EVI-ASI features to different channels, providing rich frequency domain and spatial information as input to the model. In the first part of the model, a Convolutional Neural Network (CNN) is used to progressively extract features layer by layer. Specifically, 12 samples are grouped together and passed through three convolutional layers (with kernel sizes of 32, 64, and 64, respectively), gradually capturing and refining high-level local frequency and spatial features. After feature extraction, the output enters the $F^{2}CA$ module, which utilizes a frequency channel attention mechanism based on Fast Fourier Transform (FFT) to weight the features of each frequency band and spatial channel. This mechanism highlights the contribution of key features, improving the model’s focus on important information and further enhancing the effectiveness of feature representation. Before entering the KAN module, the feature maps of the 12 samples are fused into a single comprehensive feature matrix. The KAN module captures the complex feature relationships between samples through nonlinear mapping and feature transformation, thereby improving the accuracy and robustness of emotion recognition. Finally, the fused features are passed through a classification layer to output the final emotion category prediction.

Next, this paper will describe the core modules of the model in detail: feature extraction and individual difference correction, feature mapping, $F^{2}CA$ attention mechanism, and the KAN module.

### Feature extraction and individual difference correction

(1) EEG Signal Feature Extraction

Due to the low dimensionality of the data from sparse EEG channels and the limited number of extractable features, extracting effective features from the limited channels has become a key challenge in emotional recognition research. Based on existing studies, this paper extracts frequency domain features, time-frequency domain features, and features incorporating spatial information from EEG signals, specifically including the following:

1. Power Spectral Density (PSD): Describes the power distribution of a signal in the frequency domain, calculated using the Welch method. The formula is as follows:

$$\text{PSD}=\int_{f_{1}}^{f_{2}}\text{PSD}(f)\;df$$ (1)

Where *f*1 and *f*2 represent the frequency range of the band.

2. Differential Entropy (DE): Based on the extension of Shannon’s information entropy, it is used to describe the uncertainty and complexity of continuous random variables (such as EEG signals) [20, 21]. Under the assumption of a Gaussian distribution, its calculation formula is as follows:

$$DE=\frac{1}{2}\log(2\pi e\sigma^{2})$$ (2)

Where $\sigma^{2}$ is the variance of the signal.

3. Asymmetry Index (ASI): Reflects the hemispherical asymmetry of frontal Theta rhythm power [22]. The calculation formula is as follows:

$$EVI=\log\left(\frac{Theta_{ch1}}{Theta_{ch2}}\right)$$ (3)

Where *Theta*_*ch*1_ and *Theta*_*ch*2_ represent the power of two different channels in the theta band.

4. Asymmetry Index (ASI): Used to measure the power difference between two EEG signal channels in the (α,β,γ) frequency bands. The calculation formula for ASI is as follows:

$$ASI=\frac{P_{ch1}-P_{ch2}}{P_{ch1}+P_{ch2}}$$ (4)

where and represent the power of two channels in a specific frequency band.

(2) Individual Difference Correction

In emotional recognition tasks, individual physiological signals exhibit significant differences. These differences are not only reflected in the amplitude and waveform characteristics of signals from different individuals but also in the physiological response patterns to emotional stimuli. Due to the highly personalized nature of emotional responses, directly merging data from different subjects may introduce considerable errors, thus reducing the performance and generalization ability of emotion recognition models. Therefore, to address this issue, a feature-level correction method based on individual differences is proposed, aiming to improve the accuracy of emotion recognition tasks.

This study uses the public datasets DEAP and SEED for experimentation. In these datasets, the division between baseline and experimental signals is as follows:

DEAP dataset: The total signal length is 63 seconds, with the first 3 seconds as baseline signals and the remaining 60 seconds as experimental signals. SEED dataset: The first 3 seconds of the signal are defined as baseline signals.

The baseline signals are segmented into 1-second intervals, and multiple features are extracted from each segment. Taking the DEAP dataset as an example, the 3-second baseline signal is divided into 3 segments. For a particular feature, three feature values (*F*_1_, *F*_2_, *F*_3_) are obtained from each segment. The mean value of this feature in the baseline signal, denoted as $\bar{F}$, is calculated using the following formula:

$$\bar{F}=\frac{1}{3}(F_{1}+F_{2}+F_{3})$$ (5)

Then, the features of the baseline signals and experimental signals are standardized. Considering that different features may have different units, and to preserve the relative differences between features, each feature is standardized individually. The Max-Min normalization method is used to scale the feature values to the range of [0, 1]. The calculation formula is as follows:

$$F^{\prime }=\frac{F-F_{\min}}{F_{\max}-F_{\min}}$$ (6)

Where *F* is the original feature value, and *F*_*min*_ and *F*_*max*_ are the minimum and maximum values of the feature, respectively.

After the standardization has been completed, the features are further corrected. The correction is performed using the following formula:

$$F_{\text{corrected}}=F-\bar{F}$$ (7)

Where *F*_*corrected*_ represents the corrected feature value, *F* is the feature value of the experimental signal, and $\bar{F}$ is the mean value of the baseline feature.

This correction method significantly reduces the noise introduced by individual differences by eliminating the influence of baseline features, thereby enhancing the adaptability and generalization ability of the emotion recognition model for different individuals.

### Feature mapping

**Fig 2:**
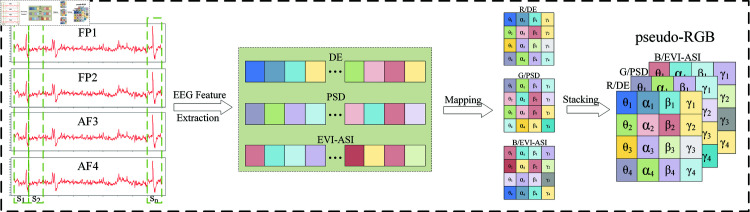
Pseudo-RGB feature matrix structure. The data preprocessing process includes segmentation, feature extraction, mapping, and stacking the Pseudo-RGB structure.

To simultaneously integrate frequency domain information and spatial features from the limited EEG channels, this study proposes a strategy based on multi-band feature extraction. Through in-depth analysis of the sparse EEG channel signals, the frequency domain characteristics and spatial distribution are maximized to improve the accuracy of emotional recognition. As shown in Fig 2, the raw EEG signals are first segmented into multiple 1-second non-overlapping time windows and processed using a sliding window method, with time segments from *S*_1_ to *S*_*n*_.

In terms of feature extraction, the DE features in the frequency bands θ, α, β and γ are first extracted from the four EEG channels (FP1, FP2, AF3 and AF4), resulting in a total of 16 DE features: $DE_{\theta 1}$ -$DE_{\theta 4}$, $DE_{\alpha 1}$ -$DE_{\alpha 4}$, $DE_{\beta 1}$ -$DE_{\beta 4}$, $DE_{\gamma 1}$ -$DE_{\gamma 4}$. Additionally, 16 PSD features are extracted, including $PSD_{\theta 1}$ -$PSD_{\theta 4}$ , $PSD_{\alpha 1}$ -$PSD_{\alpha 4}$, $PSD_{\beta 1}$ -$PSD_{\beta 4}$, $PSD_{\gamma 1}$ -$PSD_{\gamma 4}$. For spatial feature extraction, four EVI features are extracted for the θ band, namely $EVI_{FP1-FP2}$, $EVI_{AF3-AF4}$, $EVI_{FP1-AF4}$, $EVI_{AF3-FP2}$. Furthermore, 12 ASI features are extracted for the α, β and γ bands, resulting in a total of 16 spatial features. As a result, the number of DE, PSD, and EVI-ASI features is 16 each.

To fully explore the spatiotemporal characteristics of EEG signals and the intrinsic relationships between different frequency bands and features, this study designs a feature representation method based on structured mapping, as shown in Fig 2. Specifically, the 16 feature values of each feature are mapped into a 4×4 feature matrix. This mapping not only shows the distribution characteristics of the same feature across different frequency bands but also reflects the intrinsic connections between the four channels, providing important insights for modeling feature correlations.

Finally, the three 4×4 feature matrices extracted based on DE, PSD, and EVI-ASI are stacked along the Y-axis, forming a 3D feature structure—a pseudo-RGB image. The Y-axis direction captures the relationships between different features under the same frequency band and channel. This 3D feature structure is then represented as a 4D tensor with the shape (batch_size, height, width, channels) = (batch_size, 4, 4, 3). This tensor format can be directly input into a 2D CNN to extract deeper features, facilitating the comprehensive capture of multidimensional information from EEG signals in the time, space, and frequency domains.

### $F^{2}CA$ attention mechanism

Traditional global average pooling only computes the spatial average for each channel, while Fourier Transform (FT) captures global variation patterns within the input feature maps. This means that it not only extracts local information but also identifies frequency variations in the channels, potentially capturing more non-local features. Especially in tasks like emotion recognition or signal processing, frequency-domain information often holds greater significance than spatial-domain information, and Fourier Transform can highlight these frequency components. To enhance the ability of convolutional neural networks to model channel features, this paper adopts the idea of Frequency Channel Attention (FCA) [23] and proposes a Frequency Channel Attention mechanism based on Fast Fourier Transform ($F^{2}CA$). Compared to the Discrete Cosine Transform (DCT) used in FCA, $F^{2}CA$ uses FFT to convert time-domain features into frequency-domain features, making it more suitable for extracting comprehensive frequency features, especially for emotion recognition using sparse EEG channels.

**Fig 3:**
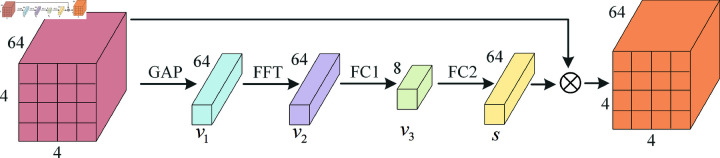
$F^{2}CA$ module structure. The process of the $F^{2}CA$ attention mechanism includes: first, extracting global features, then performing frequency domain processing, and finally generating channel attention to weight the features from different channels.

The structure of $F^{2}CA$ is shown in Fig 3 and mainly consists of three parts: global feature extraction, frequency domain processing, and channel attention generation. Given an input feature map with dimensions 4×4×64, where 4×4 represents the spatial dimensions of the feature map and 64 indicates the number of channels. First, global average pooling is applied to the spatial dimensions of each channel of the input feature map, producing a global descriptor vector $v_{1}$ of length 64. The formula is as follows:

$$v_{1}=\frac{1}{H\times W}\sum_{i=1}^{H}\sum_{j=1}^{W}x_{ijc}$$ (8)

Here, *x*_*ijc*_ represents the value of the input feature map at channel (i, j) and position *c* . Next, to further capture the frequency domain relationships between global features, a Fourier transform is applied to $v_{1}$ .The Fourier transform converts the input from the time domain to the frequency domain, extracting the frequency components of the signal. The specific operation is as follows:

$$\text{freg}=\text{Re}(\text{FFT}(v_{1}))$$ (9)

In the above equation, *FFT* represents the Fast Fourier Transform (FFT), and *Re* denotes taking the real part of the Fourier transform result. The purpose of this is to analyze the impact of different frequency components in the frequency domain, thereby enhancing the global representation ability of the features. Then, the frequency domain information *freg* undergoes two fully connected (FC) operations (FC1 and FC2), which are used for channel compression and reconstruction, respectively. FC1 compresses the channel dimension using the ratio parameter (default is 8), while FC2 restores the channel dimension and generates the channel attention weights through the Sigmoid activation function:

$$s=\sigma (W_{2}\cdot \text{ReLU}(W_{1}\cdot \text{freg}))$$ (10)

Here, *W*_1_ and *W*_2_ represent the weight matrices of the fully connected layers, σ denotes the Sigmoid function, and ReLU is the activation function. The final weight vector *s* has a size of 64. Lastly, the generated channel attention weights *s* are multiplied with the input feature map using a broadcasting mechanism, producing the weighted output feature map:

$$y_{ijc}=x_{ijc}\cdot s_{c}$$ (11)

Here, *y*_*ijc*_ represents the feature map adjusted by the channel attention mechanism, and *s*_*c*_ is the attention weight for the *c* -th channel. Through the Fourier transform, $F^{2}CA$ is able to capture more frequency component information, thereby enhancing the model’s ability to perceive global patterns.

### KAN module

To enhance the nonlinear fitting capability of the emotion recognition model, this paper introduces the Kolmogorov-Arnold Network (KAN) [24]. Based on the Kolmogorov-Arnold representation theorem, this layer can decompose multivariable functions into combinations of several univariable functions, thereby effectively improving the model’s ability to extract features from complex EEG signals.

**Fig 4:**
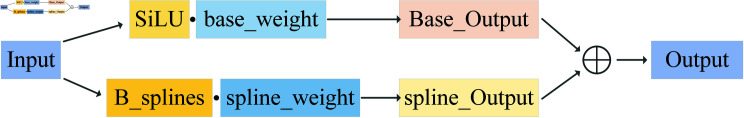
KAN calculation flowchart. The computation of KAN is divided into two parts: baseline output and spline interpolation output.

As shown in Fig 4, the KAN computation is divided into two parts: baseline output and spline interpolation output. First, the input feature $X\in {\mathbb{R}}^{N\times d_{in}}$ undergoes a linear transformation to obtain the baseline output. The specific calculation formula is as follows:

$$\text{BaseOutput}=\sigma (X)\cdot W_{\text{base}}$$ (12)

Here, *X* represents the input feature matrix, $W_{\text{base}}\in {\mathbb{R}}^{d_{in}\times d_{out}}$ is the trainable baseline weight matrix, and σ(·) is the SiLU nonlinear activation function.

Next, KAN performs nonlinear mapping based on the B-spline interpolation mechanism. By constructing a spline interpolation grid, the input features undergo spline interpolation to produce the spline output. The calculation process of B-spline interpolation is as follows:

$$S(X)=\sum_{k=0}^{k}B_{k}(X)\cdot W_{\text{spline},k}$$ (13)

Here, *B*_*k*_(*X*) represents the B-spline basis functions, $W_{\text{spline},k}$ is the trainable weight matrix associated with the spline basis functions *B*_*k*_(*X*), and *K* denotes the order of the spline. Spline interpolation allows for the nonlinear transformation of input features, capturing complex patterns in EEG signals. To further enhance the model’s feature representation ability, the KAN layer introduces an adaptive scaling mechanism. This mechanism uses a learnable scaling factor Spline Scaler to weight the spline output, adjusting the importance of the features. The calculation formula is as follows:

Spline Output=S(X)·Spline Scaler (14)

Where $\text{Spline Scaler}\in {\mathbb{R}}^{d_{1}\times d_{out}}$ is a trainable parameter used to adjust feature weights.

The final output of the KAN layer is the weighted sum of the baseline output and the spline output, that is:

Output=Base Output+Spline Output (15)

Through this design, the KAN layer can simultaneously capture both linear relationships and nonlinear variations in the input features, enabling more effective handling of sparse EEG data. In emotion recognition tasks, this multi-level feature representation significantly enhances the model’s performance.

KAN enhances the model’s adaptability in extracting complex EEG signals, especially when dealing with sparse channels. By combining baseline output and spline interpolation output, KAN effectively captures the correlation between local and global features, thereby improving the accuracy of emotion recognition. Additionally, the adaptive scaling mechanism ensures the model’s flexibility in processing features from different frequencies and channels.

## Experimental design

### Datasets and data preprocessing

The DEAP dataset is a widely used multimodal public dataset in the field of emotion recognition, developed by Koelstra *et al*. [2]. This dataset contains EEG and other physiological signals from 32 subjects who watched 40 music video clips. During the experiment, participants wore a 32-channel electrode cap adhering to the international 10-20 system, with EEG signals sampled at 512 Hz and downsampled to 128 Hz for processing. Each video clip lasted for 1 minute, and after watching, participants rated the videos on four dimensions: Valence, Arousal, Dominance, and Liking, using a scale from 1 to 9. This paper uses the data version filtered with a 4-45 Hz bandpass filter, with EOG artifacts and other noise signals removed. The EEG channels FP1, FP2, AF3, and AF4 were selected. A binary classification task was performed for the Arousal and Valence dimensions, and the effectiveness of the proposed model was validated in a four-class task based on the joint Arousal-Valence dimensions.

The SEED dataset [25] includes EEG signals from 15 subjects (8 females, 7 males) while watching emotion-inducing videos. Each participant watched 15 video clips, each approximately 4 minutes long. The experiment was conducted three times, with a one-week interval between sessions, resulting in a total of 45 EEG data recordings. EEG signals were recorded using a 62-channel electrode cap with a sampling rate of 1,000 Hz, then downsampled to 200 Hz, with eye-movement artifact removal and 4-50 Hz bandpass filtering applied. This paper selects the FP1, FP2, AF3, and AF4 EEG channels, applies a 4-45 Hz bandpass filter, and then evaluates the model’s effectiveness on a three-class task with Positive, Negative, and Neutral emotional categories.

### Experimental parameter settings

All experiments were conducted in a unified hardware and software environment, with consistent dataset partitioning methods and parameter settings, to ensure the comparability and reliability of the results. The hardware environment includes a Mechanical Revolution laptop equipped with an Intel Core i5-12450H @ 2.00GHz processor and an Nvidia GeForce RTX 3060 GPU. The software environment is based on the Windows 11 operating system, with Python 3.8 and TensorFlow 2.10.0 for model development and training.

The CNN-KAN-$F^{2}CA$ emotion recognition model proposed in this paper uses the minimization of classification cross-entropy and an L2 regularization term (with a regularization coefficient of 0.001) as the loss function. The Adam optimizer is used for model optimization, with a learning rate of 0.0005 and a batch size of 512. The number of training epochs is set to 200. To prevent overfitting, Dropout is applied after multiple convolutional layers, with Dropout rates of 0.5, 0.4, and 0.3, respectively. Dropout is also applied in the fully connected layers (with a value of 0.5). During the experiment, a random seed of 2024 is used to shuffle the data, and 10-fold cross-validation is employed to divide the dataset into 10 subsets. Nine subsets are used for training, and the remaining one is used for testing, with no repetition.

### Evaluation metrics

In this paper, Accuracy, Precision, Recall, and F1 Score are used as the primary evaluation metrics for model performance. Precision, Recall, and F1 Score are calculated using the weighted average (Weighted Avg) to fully account for the impact of class imbalance on the model’s performance. Below are the definitions and calculation methods for each metric:

$$\text{Accuracy}=\frac{TP+TN}{TP+TN+FP+FN}$$ (16)

$$\text{Precision}=\frac{TP}{TP+FP}$$ (17)

$$\text{Recall}=\frac{TP}{TP+FN}$$ (18)

$$\text{F1 Score}=2\times \frac{\text{Precision}\times \text{Recall}}{\text{Precision}+\text{Recall}}$$ (19)

Where TP (True Positive) refers to the number of correctly identified positive samples, TN (True Negative) refers to the number of correctly identified negative samples, FP (False Positive) represents the number of negative samples incorrectly predicted as positive (false alarm), and FN (False Negative) refers to the number of positive samples incorrectly predicted as negative (missed detection).

## Results and analysis

### Model ablation experiment

**Fig 5:**
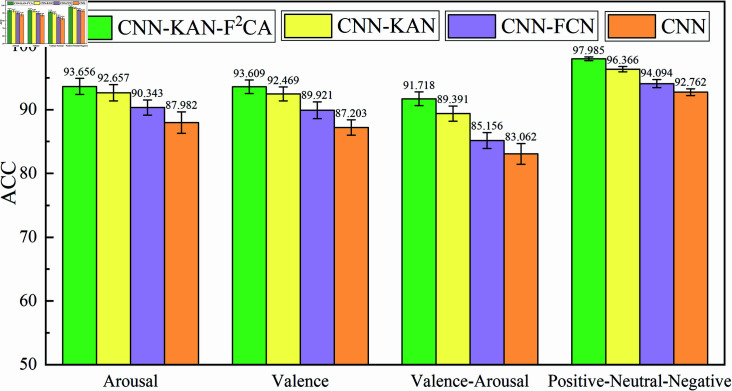
EEG emotion recognition accuracy of different models on DEAP and SEED datasets.

**Fig 6:**
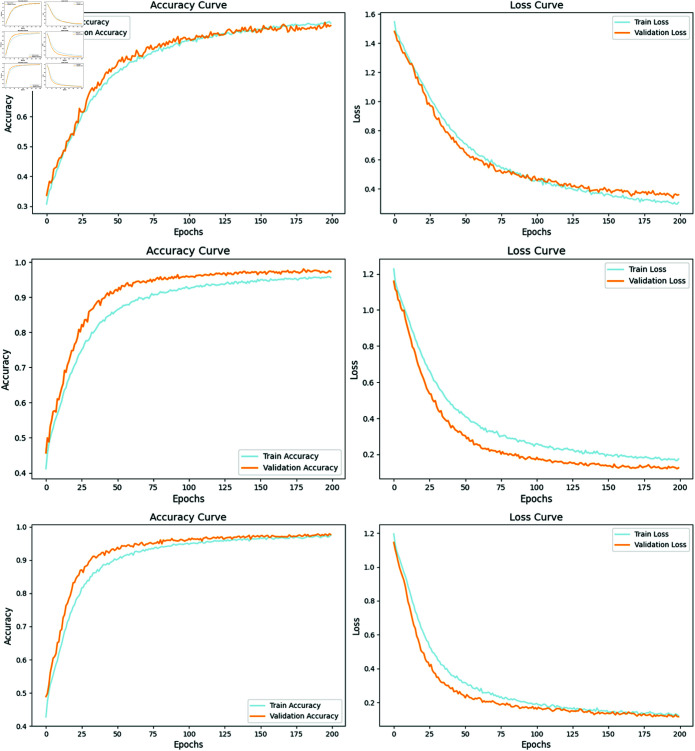
Accuracy and loss curves of the CNN-KAN-$F^{2}CA$ model on DEAP (top), SEED without hyperparameter optimization (middle), and SEED with the dropout rate of the fully connected layer reduced to 0.3 (bottom).

To comprehensively assess the impact of each module on the overall performance of the model, this study conducted an ablation experiment on two public datasets: DEAP and SEED. On the DEAP dataset, data from 32 subjects were merged, while on the SEED dataset, data from three recordings of 15 subjects were merged to construct a cross-subject dataset. By systematically removing different modules and comparing their corresponding classification performance, the contribution and effectiveness of each module in the model were validated. Specifically, the study performed in-depth analysis on the following model structures: baseline CNN model, CNN-FCN model, CNN-KAN model, and the CNN-KAN-$F^{2}CA$ model incorporating attention mechanisms. Fig 5 shows the performance of these four models on the DEAP and SEED datasets, and a detailed comparison of their accuracy across different tasks. Fig 6 presents the accuracy and loss curves of the CNN-KAN-$F^{2}CA$ model when the training, validation, and testing sets are split in an 8:1:1 ratio.

As shown in Fig 5, compared to the basic CNN network model, the CNN-KAN-$F^{2}CA$ model demonstrated significant improvements in classification accuracy on both the DEAP and SEED datasets. Specifically, on the DEAP dataset, the accuracy for the four-class task, wakefulness binary classification task, and valence binary classification task increased by 8.656%, 5.674%, and 6.406%, respectively. On the SEED dataset, the accuracy in the three-class task improved by 5.223%. These results indicate that the CNN-KAN-$F^{2}CA$ model significantly enhanced the classification performance by incorporating the $F^{2}CA$ module and the KAN module.

Fig 6 presents the training and validation accuracy and loss curves of the CNN-KAN-$F^{2}CA$ model on the DEAP (top) and SEED (without hyperparameter optimization, middle) datasets. It can be observed that accuracy increases on both datasets, indicating that the model successfully learns effective features. Meanwhile, training and validation loss decrease with more training epochs, showing continuous optimization and error reduction.

It is important to note that our hyperparameters were tuned on the DEAP dataset and directly applied to SEED without separate optimization. As a result, validation accuracy on SEED is higher than training accuracy, which may be influenced by data distribution, task complexity, and regularization strategies (BN layers, Dropout, L2). Nevertheless, both training and validation loss continue to decrease without signs of increase.

Additionally, when the dropout rate of the fully connected layer was reduced from 0.5 to 0.3 (bottom figure), training accuracy improved further and became closer to validation accuracy. This suggests that the observed effect is mainly due to the strength of regularization rather than overfitting. Overall, the model demonstrates stable performance on both training and validation sets, with no significant signs of overfitting.

As shown in Table 1, in the four-class task on the DEAP dataset, the results of the model ablation experiment indicate that the CNN-KAN-$F^{2}CA$ model, which incorporates the $F^{2}CA$ module, achieved an accuracy improvement of 2.33% compared to the CNN-KAN model. Although the number of parameters increased by about 0.21% and the inference time increased by about 0.02 seconds, the $F^{2}CA$ module effectively enhanced the model’s ability to capture global frequency information, thereby improving classification performance. In the comparison between CNN-KAN and CNN-FCN, the introduction of the KAN module led to an increase in parameters by about 47.0% and an increase in inference time by about 0.02 seconds, but the accuracy improved by 4.23%. This proves that the KAN module significantly enhanced the model’s capacity to learn complex local features adaptively. Moreover, compared to the baseline CNN model, CNN-KAN with the KAN module achieved an accuracy improvement of 6.33%. Despite the increase in the number of parameters and training time, the model’s advantage in handling complex emotion classification tasks is evident.

**Table 1:** Results of model ablation experiments.

Dataset	Method	P/R/F1 (%)	ACC (%)	Params	Inference Time (s)
DEAP	CNN-KAN-F2CA	91.72/91.72/91.72	91.72	519,756	0.18
	CNN-KAN	89.43/89.39/89.39	89.39	518,660	0.16
	CNN-FCN	85.24/85.16/85.15	85.16	352,772	0.15
	CNN	83.1/83.06/83.04	83.06	320,388	0.14
SEED	CNN-KAN-F2CA	97.99/97.99/97.99	97.99	519,756	0.28
	CNN-KAN	96.97/96.37/96.37	96.37	518,660	0.25
	CNN-FCN	94.1/94.09/94.09	94.09	352,772	0.23
	CNN	92.76/92.76/92.76	92.76	320,388	0.22

Overall, the KAN and $F^{2}CA$ modules effectively enhanced the model’s ability to capture both complex local features and global features, significantly improving the accuracy of emotion classification tasks. Although the computational cost increased, the performance gains make the model highly valuable for applications requiring high accuracy.

The experimental results demonstrate that the CNN-KAN-$F^{2}CA$ model proposed in this study performs exceptionally well on both the DEAP and SEED datasets, significantly improving emotion recognition accuracy. By incorporating the $F^{2}CA$ and KAN modules, the model can more effectively extract and integrate features, leading to superior performance in complex tasks. Despite its higher computational cost, the performance improvement indicates that this model is suitable for applications that require high accuracy. Future research could focus on further optimizing the model’s computational efficiency to enable broader adoption in practical applications.

### Feature channel ablation experiment

In this experiment, feature channel ablation was conducted on the proposed CNN-KAN-$F^{2}CA$ model using the DEAP and SEED public datasets. For the DEAP dataset, data from 32 subjects were combined; for the SEED dataset, three recordings from 15 subjects were merged to create a cross-individual dataset. By removing a particular channel from the pseudo-RGB channels, the changes in emotion recognition accuracy were observed, allowing for an assessment of the role of each feature channel in the model.

In this experiment, four types of features—DE, PSD, EVI, and ASI—were extracted and mapped into three-channel pseudo-RGB images for the emotion recognition task. The model was evaluated using 10-fold cross-validation, and the impact of different feature channel combinations on emotion recognition accuracy was analyzed through comparison experiments.

**Fig 7:**
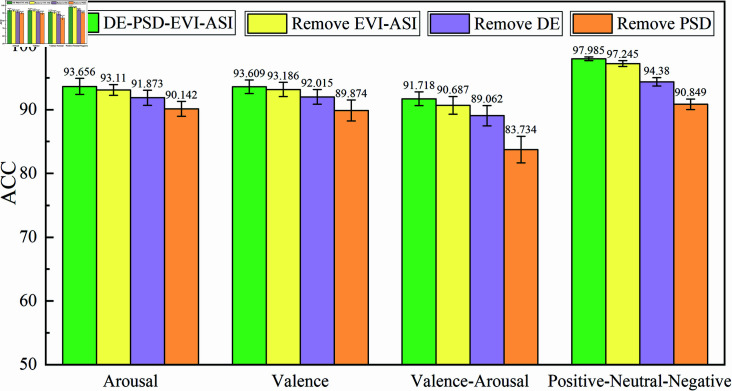
Accuracy of CNN-KAN-$F^{2}CA$ model under different feature channel combinations.

The results of the feature channel ablation experiment are shown in Fig 7 and analyzed as follows:

- **DE-PSD-EVI-ASI (Baseline Experiment):** The combination of DE, PSD, EVI, and ASI features achieved the best emotion recognition accuracy. In the four-class task on the Valence-Arousal dimension, the accuracy reached 91.718%; in the binary classification tasks on Arousal and Valence dimensions, the accuracies were 93.656% and 93.609%, respectively. For the three-class task on the SEED dataset, the accuracy was 97.985%.
- **DE-PSD (Remove EVI and ASI Features):** Removing the EVI and ASI spatial features caused a decrease in emotion recognition accuracy. In the four-class task on the Valence-Arousal dimension, the accuracy dropped by 1.031%; in the binary classification tasks on Arousal and Valence dimensions, the accuracies dropped by 0.546% and 0.423%, respectively. For the three-class task on the SEED dataset, the accuracy dropped by 0.74%.
- **PSD-EVI-ASI (Remove DE Feature):** Removing the DE feature led to a further decline in the model’s overall performance. The accuracy on the Valence-Arousal dimension decreased by 2.656%, and the accuracies on the Arousal and Valence dimensions dropped by 1.783% and 1.594%, respectively. For the three-class task on the SEED dataset, the accuracy dropped by 3.605%.
- **DE-EVI-ASI (Remove PSD Feature):** The accuracy dropped most significantly when the PSD feature was removed. On the Valence-Arousal dimension, the model’s accuracy was only 83.734%, a 7.984% decrease compared to the full feature channel combination. The accuracies on Arousal and Valence dimensions dropped by 3.514% and 3.735%, respectively. For the three-class task on the SEED dataset, the accuracy dropped by 7.136%. This demonstrates that the PSD feature has a crucial impact on the model’s performance.

#### Analysis of feature ablation results.

The feature ablation experiment shows that the complete combination of all features (DE, PSD, EVI, and ASI) yields the best emotion recognition results, verifying the effectiveness of the multi-frequency feature extraction strategy. Notably, the DE and PSD frequency domain features played an essential role in improving model performance. Removing these two features significantly reduces the model’s emotion recognition ability. In contrast, removing spatial features (EVI and ASI) also leads to a decrease in accuracy, but the impact is comparatively smaller.

From the feature ablation experiment, it can be concluded that the frequency domain features (DE and PSD) make a particularly significant contribution to emotion recognition, while spatial features also enhance the model’s performance to some extent. Therefore, the feature extraction strategy proposed in this paper, by combining frequency domain features with spatial features, maximizes the information within the limited EEG channels and effectively improves emotion recognition accuracy.

### Subject-dependent experiments

"Subject-dependent experiments" refer to the scenario in emotion recognition tasks where both training and testing of the model are based on data from individual subjects. Since there are significant differences in EEG signal characteristics across individuals, subject-specific emotional patterns may influence the model’s performance. This is especially true in cross-subject emotion recognition tasks, where individual differences could lead to a decrease in the model’s generalization ability. In this study, we address these individual differences by using the CNN-KAN-$F^{2}CA$ model to compare the performance of the model with single-subject data versus multiple-subject data. Unlike the model ablation experiment (where data from all subjects were merged for training and testing), this experiment focuses on training and testing with single-subject data, aiming to evaluate the model’s emotion recognition ability for specific individuals.

The experimental tasks include binary classification, three-class classification, and four-class classification on the DEAP and SEED datasets. By comparing the experimental results from single-subject and multiple-subject datasets, we can better understand how individual differences impact emotion recognition performance and explore how the model’s generalization ability and recognition accuracy change with varying dataset sizes.

#### Analysis and Results Discussion.

In the single-subject dependent experiment, as shown in Table 2, the accuracy of the DEAP dataset across different dimensions is as follows:

- **Valence Dimension**: The average accuracy of subjects was 90.03%, with a maximum of 95.5% (subjects 03, 29) and a minimum of 84.5% (subject 02).
- **Arousal Dimension**: The average accuracy was 91.33%, with a maximum of 97.5% (subjects 03, 18) and a minimum of 83% (subject 02).
- **Valence-Arousal Dimension**: The average accuracy was 89.16%, with a maximum of 96% (subject 25) and a minimum of 81% (subject 30).
- **SEED Three-Class Classification:** The average accuracy was 97.45%, with a maximum of 98.52% (subject 14) and a minimum of 95.56% (subject 01).

**Table 2:** Results of subject-dependent experiments.

Subject	DEAP–Accuracy (%)			SEED–Accuracy (%)
	Arousal	Valence	Arousal-Valence	
01	95.5	91	92	95.56
02	83	88	82.5	97.11
03	97.5	95.5	95.5	97.85
04	90.5	87	87	96.44
05	83.5	85.5	83.5	97.04
06	91	93.5	92.5	98.15
07	96	94.5	94.5	98.37
08	90.5	89	87.5	98.15
09	89.5	87	84	98
10	92.5	91.5	90	97.33
11	88.5	89	82.5	97.41
12	94	90.5	93.5	96.67
13	89	90.5	89	97.33
14	86.5	87	85	98.52
15	89	93	89	97.85
16	95	91	92	-
17	85.5	84.5	84.5	-
18	97.5	89	91	-
19	90.5	92.5	90	-
20	95	92.5	91	-
21	97	89	92.5	-
22	92.5	87.5	93	-
23	93	93	91	-
24	95	86	89.5	-
25	96	94.5	96	-
26	93.5	93	94	-
27	90.5	93	87	-
28	89.5	85	83	-
29	93	95.5	94.5	-
30	85	85.5	81	-
31	87.5	85.5	88	-
32	90	91.5	87	-
Average	91.328	90.031	89.156	97.452

However, when the subject data were merged, the model’s accuracy significantly increased. For the DEAP dataset:

- **Valence Dimension**: The accuracy was 93.609%.
- **Arousal Dimension**: The accuracy was 93.656%.
- **Valence-Arousal Dimension**: The accuracy was 91.718%.
- **SEED Three-Class Classification:** The accuracy was 97.985%.

These results suggest that the overall accuracy when merging subject data is significantly higher than the results from single-subject dependent experiments. The difference is mainly attributed to the following factors:

1. **Data Quantity and Model Generalization Ability**: After merging data from all subjects, the training data volume significantly increases, allowing the model to be exposed to more samples and learn more common features for emotion recognition. Compared to training with data from a single subject, the merged dataset enables the model to better capture global emotional patterns across individuals, reducing the risk of overfitting when individual data is insufficient. In single-subject training, the smaller dataset makes the model prone to overfitting, leading to lower classification accuracy for some subjects.
2. **Impact of Individual Differences**: Since the EEG signal characteristics vary significantly across different subjects, the accuracy of the model is strongly affected by individual differences during training. For example, some subjects may achieve high accuracy (e.g., subjects 03 and 29 with a 95.5% accuracy in the Valence dimension), while others may perform poorly (e.g., subject 02 with 84.5% accuracy). However, after merging the datasets, the individual differences are "smoothed out," and the model focuses more on common emotional features across individuals, improving robustness. The CNN-KAN-$F^{2}CA$ model, by training based on short temporal segments, reduces the dependency on overall EEG features of individual subjects, effectively diminishing the negative impact of individual differences.
3. **Model Robustness and Increased Diversity**: Merging subject data not only increases the sample size but also enhances the model’s adaptability to diverse EEG signals. The differences in EEG signals from different subjects provide more training samples, enabling the model to learn both common and differentiating features in the emotion recognition task. By training on a more diverse set of EEG segments, the CNN-KAN-$F^{2}CA$ model exhibits stronger robustness for cross-subject emotion recognition.
4. **Summary of Experimental Results**: The results of the experiment demonstrate that individual differences significantly affect the performance of EEG-based emotion recognition models. During training with data from a single subject, the model shows large variability in performance, with some subjects achieving much higher accuracy than others. However, after merging data from all subjects, the overall accuracy significantly improves, especially in the Valence-Arousal dimension, where the emotion recognition accuracy reaches 91.718%, notably higher than the single-subject dependent results. By training with discretized EEG segments, the CNN-KAN-$F^{2}CA$ model effectively captures the short-term emotional features in each segment, reducing the impact of individual overall features on model performance, thus showing better cross-subject emotion recognition robustness.

## Discussion

### Comparison with similar studies

As shown in Table 3, we compared the proposed CNN-KAN-$F^{2}CA$ model with several representative emotion classification models in recent years, using the DEAP public dataset. The results indicate that, with only 4 sparse EEG channels, the CNN-KAN-$F^{2}CA$ model achieved impressive performance in emotion classification tasks on the DEAP dataset. Specifically, for the classification of emotion in terms of arousal and valence, the model’s accuracy reached 93.656% and 93.609%, respectively. These results demonstrate that, even with fewer EEG channels, the model can achieve performance comparable to, or even exceeding, many existing multi-channel methods. This highlights the significant advantages of the CNN-KAN-$F^{2}CA$ model in terms of data efficiency, computational efficiency, and overall performance.

**Table 3:** Comparison of emotion classification model performance on the DEAP dataset.

	Year	Channel	Valence-Arousal	Arousal	Valence
**Jie [3]**	2014	5	-	79.11	64.47
**Mohammadi [4]**	2017	10	-	86.75	84.05
**Mert [17]**	2018	18	-	75%	72.87%
**Bazgir [18]**	2018	10	-	91.3%	91.1%
**Gonzalez [19]**	2020	14	-	83.12	76.78
**Yin [12]**	2021	32	-	90.60	90.45
**Liu [13]**	2023	32	92.92	94.91	94.53
**Xin [26]**	2023	32	-	96.43%	96.30%
**Hou [27]**	2023	32	93.56%	96.82%	96.89%
**Dong [28]**	2024	32	-	94.31%	93.12%
**Ours**	2024	**4**	**91.718**	**93.656**	**93.609**

When compared with some classic models, such as those by Yin [11] and Liu [12], which achieved over 90% accuracy with 32 EEG channels, our approach performs similarly, or even better, with only 4 channels, emphasizing its advantages in computational efficiency and emotion recognition accuracy.

### Limitations of the method and future work

Despite achieving good emotion recognition results on public datasets like SEED and DEAP, there are still some limitations and challenges in real-world applications:

1. **Non-End-to-End Classification Method and High Computational Load**: The current model adopts a manual feature extraction method, which first extracts features from EEG signals, then inputs these features into a classification model for emotion recognition. While this method is effective in capturing emotional information from EEG signals, it is not an end-to-end learning process. In real-world applications, this means multiple processing steps are required, including feature extraction, feature selection, and dataset construction, all of which contribute to a high computational load. The manual design and computation for each step increase the overall computational burden, limiting the model’s applicability in scenarios with high real-time requirements. Future research could explore transforming the model into an end-to-end learning approach, enabling the model to automatically learn emotional features directly from raw EEG signals, thus reducing intermediate steps and improving processing efficiency.
2. **Noise and Equipment Influence in Real-World Environments**: In practical applications, EEG signals are susceptible to noise, acquisition equipment, individual differences, and electrode contact quality, which may make EEG signals insufficiently stable and accurate in dynamic and complex real-world environments. For example, when signal quality is poor, the model’s performance may significantly degrade. In experiments with public datasets, standardized acquisition equipment and relatively good data quality are typically used, but in real-world settings, device diversity and individual differences could lead to fluctuations in emotion recognition performance. To enhance the model’s applicability in real environments, future research could combine peripheral physiological signals (such as heart rate, galvanic skin response (GSR), etc.) for multimodal emotion recognition. These peripheral signals could provide additional dimensions of information for emotion recognition, helping to compensate for the shortcomings of EEG signals and thus improving classification accuracy and robustness.

## Conclusion

In this study, we proposed a new sparse-channel EEG emotion recognition model, CNN-KAN-$F^{2}CA$, which addresses the challenges of redundant information in full-brain channel methods and the insufficient accuracy faced by current few-channel emotion recognition approaches. By innovatively combining feature mapping, the $F^{2}CA$ attention mechanism, and the KAN network, the model can extract effective emotional features within limited EEG channels while enhancing cross-subject emotion recognition robustness.

Experimental results on the SEED and DEAP datasets show that the CNN-KAN-$F^{2}CA$ model performs excellently in emotion classification tasks. In the merged subject dataset experiment, the accuracy of the SEED three-class classification task reached 97.985%, while the accuracy of the DEAP four-class classification task was 91.718%. In the subject-dependent experiment, the average accuracy for the SEED three-class task was 97.45%, and for the DEAP four-class task, it was 89.16%.

Although the model uses only 4 EEG channels, experimental results show that its emotion recognition accuracy is close to that of multi-channel models. At the same time, the training strategy based on short temporal segments effectively reduces the impact of individual differences on emotion recognition performance, enhancing the model’s robustness and generalization ability. In cross-subject tasks, after merging training data, the model’s performance was further improved, demonstrating its significant advantages in handling individual differences.
